# Chronic post-dural puncture headache–a serious and underrated complication following lumbar puncture: a cohort study

**DOI:** 10.3389/fneur.2024.1493303

**Published:** 2024-11-29

**Authors:** Luisa Mona Kraus, Levin Häni, Amir El Rahal, Ioannis Vasilikos, Mateo Tomas Fariña Nuñez, Florian Volz, Horst Urbach, Niklas Lützen, Christian Ulrich, Jürgen Beck, Christian Fung

**Affiliations:** ^1^Department of Neurosurgery, Medical Center, University Hospital Freiburg, Freiburg im Breisgau, Germany; ^2^Department of Neurosurgery, School of Medicine, Klinikum Rechts Der Isar, Technical University Munich, Munich, Germany; ^3^Department of Neurosurgery, Inselspital Bern, University Hospital Bern, Bern, Switzerland; ^4^Spine Center, Schulthess Klinik, Zurich, Switzerland; ^5^Department of Neuroradiology, Medical Center, University Hospital Freiburg, Freiburg im Breisgau, Germany; ^6^Department of Neurosurgery, Lindenhofspital of Bern, Bern, Switzerland

**Keywords:** postdural puncture headache (PDPH), chronic headache, lumbar puncture (LP), peridural anesthesia, socioeconomial impact

## Abstract

**Background:**

Post-dural puncture headache (PDPH) is still mostly regarded a minor complication after lumbar puncture. In the International Classification of Headache Disorders (ICHD)-3 headaches lasting longer than 14 days or persisting after epidural blood patch (EBP) are not even considered. We illustrate that there may be many patients with persisting headaches and a large disease burden.

**Methods:**

In a retrospective, single center analysis from 04/2018 to 03/2022 we assessed patients with a dural puncture and orthostatic headache of >14 days duration, resistant to one or more EBPs. Socioeconomic factors and individual patient history were assessed by a specifically designed questionnaire.

**Results:**

We included 30 patients with a mean age of 36.4 (±10.6) years. The median duration of acute inpatient care was 31 (Interquartile ratio (IQR) = 32) days and of sick leave 381 (IQR = 666.3) days. Patients consulted a median of 5 (IQR = 6.5) different physicians/ institutions due to chronic post-dural puncture headache (cPDPH). All patients reported major negative impact of cPDPH on their social and work life.

**Conclusion:**

Despite long hospitalizations and a profound impairment of social and work lives cPDPH was neglected and underrated in all patients. We conclude that cPDPH needs to be considered and might be an underreported, severe condition which requires further prospective studies.

## Introduction

Following lumbar puncture (LP), post-dural puncture headaches are considered a rare and minor complication. The individual impact on patients’ personal lives is largely neglected, patients feel “humilated and let down by the world” (1). Accordingly, the International Classification of Headache Disorders (ICHD) describes post-dural puncture headaches as occurring within 5 days after lumbar puncture and remitting spontaneously within 14 days or after epidural blood patch (EBP), whereas the possibility of chronification is not even considered (2). The overall incidence of post-dural puncture headache is up to 36%, and more likely in younger patients (3, 4). While symptoms are commonly defined as transient, we want to describe patients suffering from persistent, post-dural puncture headaches with serious consequences.

Persisting symptoms may be subsumed as chronic post-dural puncture headache (cPDPH) and may go undiagnosed from weeks to months and even years (5–7).

We define cPDPH as a headache that lasts longer than 14 days after lumbar puncture and does not remit spontaneously or after one EPB. We aim to draw attention to these patients and to assess the burden of disease and the socioeconomic impact of cPDPH in a retrospective cohort study.

## Methods

We conducted a retrospective, observational cohort study. We obtained approval from the local ethics committee of the Albert-Ludwigs-Universität Freiburg, for this study (22-1383-S1-retro).

### Patient population

We screened all patients at the Department of Neurosurgery, Medical Center, University Hospital Freiburg between 04/2018 and 03/2022 that were treated for orthostatic headache after intentional or accidental dural puncture. Inclusion criteria were:

- History of either (i) diagnostic lumbar puncture (ii) peridural anesthesia (PDA) (iii) epidural peri-radicular therapy (PRT) or spinal-cord-stimulation (SCS-) electrode placement
- Symptom onset and characteristics according to post-dural puncture headache as defined in the ICDH-3 (2)
- Symptom persistence >14 days, or
- Symptom persistence despite an EBP at our department

In patients with a history of peridural anesthesia, periradicular therapy or spinal cord stimulation, procedural documentation of accidental dural puncture was not a prerequisite.

Exclusion criteria were:

- Headache as the primary reason for diagnostic lumbar puncture
- Low CSF pressure headache related to other diagnosis such spontaneous intracranial hypotension or postoperative CSF leak

### Data analysis and statistics

We assessed hospital records, prior medical history, as well as imaging data. Symptom duration was defined as the period from symptom onset to first presentation at our department. We calculated length of hospital stay as the sum of all days of hospital treatments related to the post-dural puncture treatment. Number of EPBs included EBPs performed at external hospitals and at our department.

Socioeconomic factors were assessed by a specifically designed questionnaire, which was sent to all included patients (Supplementary material). Patients were asked to describe the impact of their condition on their social life, relationships and working status. Patients were clinically followed up until August 2022.

Descriptive statistics including calculation of the mean and standard deviation for normally distributed, as well as median and interquartile ranges for skewed data were applied using IBM^®^ SPSS statistics Version 27. Normal distribution was assessed graphically using boxplots and analytically using the Shapiro–Wilk test. We employed the Mann–Whitney *U* test to assess the differences between independent groups, as it is particularly suitable for non-normally distributed data and ordinal variables.

## Results

**Figure 1:**
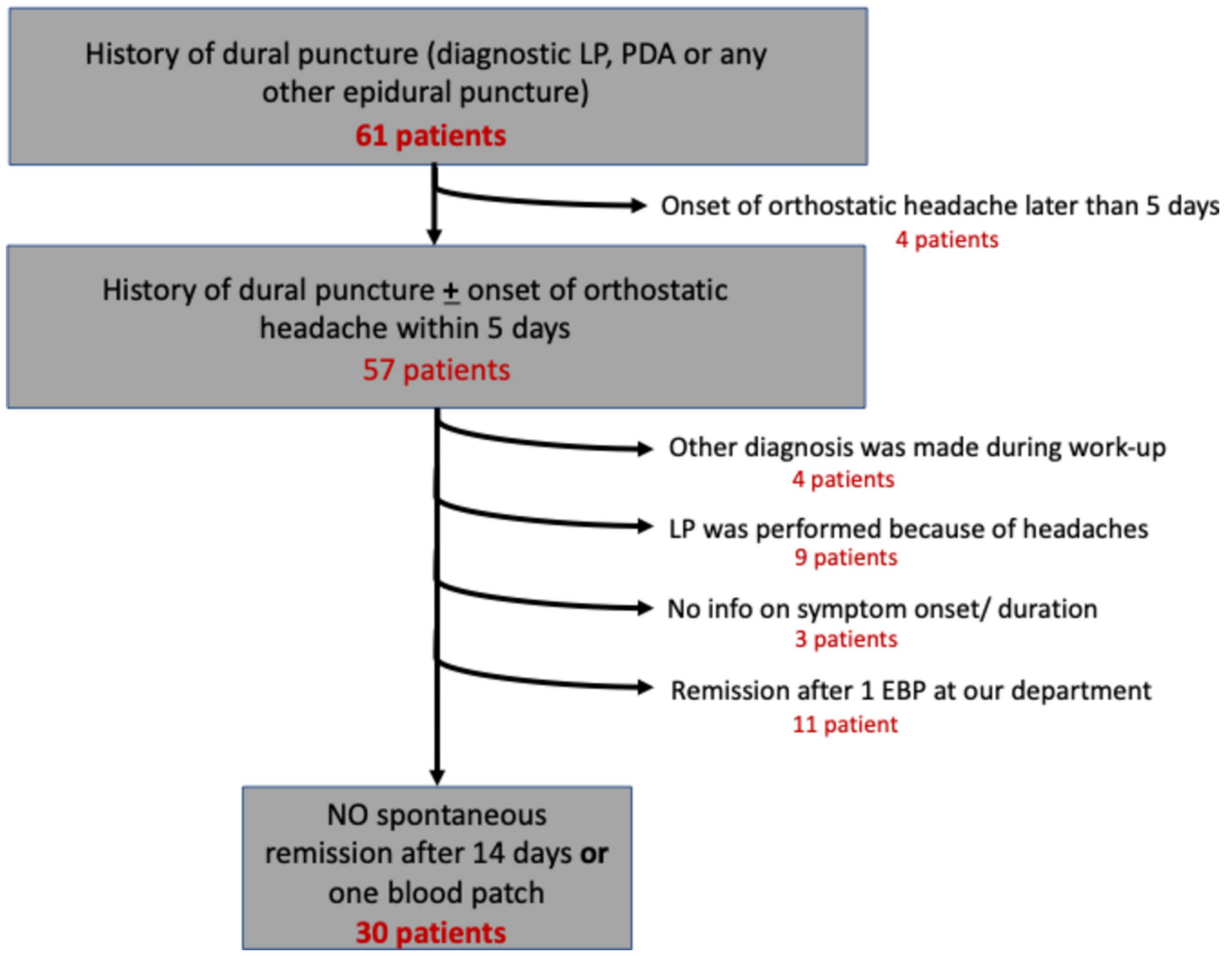
Flow chart displaying patient selection.

Between 04/2018 and 03/2022, 61 patients with post dural puncture headache were treated at our institution. A total of 30 patients (Table 1) fulfilled the inclusion criteria of cPDPH and were further analyzed in this study (Figure 1).

**Table 1:** Demographical and clinical data of study population.

		All *n* = 30	LP *n* = 16	PDA *n* = 11	Other *n* = 3
Mean age at first presentation (years +/− SEM)		36.4 (+/− 10.6)	39,4 (+/−3.2)	31.3 (+/−1.5)	34.0 (+/−12)
Female		23 (76.6%)	11 (68.8%)	11 (100%)	2 (66.7%)
Male		7 (23.4%)	5 (31.2%)	0	1 (33.3%)
Type of procedure					
Diagnostic LP		16 (53.3%)	–	–	–
PDA		11 (36.7%)			
Other		3 (10%)			
Symptoms	Orthostatic headache	30 (100%)	16 (100%)	11 (100%)	3 (100%)
	Nausea	14 (46.7%)	7 (39.8%)	5 (45.5%)	2
	Tinnitus	8 (26.7%)	3 (18.8%)	4 (36.5%)	1
	Dizziness	15 (50%)	8 (50%)	5 (45.5%)	2
	Neck stiffness	9 (30%)	6 (37.5%)	3 (27.3%)	0
Subdural hematomas		4 (13.3%)	2 (12.5%)	2 (18.2%)	0
Surgery for subdural hematoma		1 (3.3%)	1 (6.3%)	0	0
Median symptom duration at initial presentation (months) (IQR)		3.3 (11.25)	3 (7)*	5 (71)*	13 (13)
Median number of EBPs (IQR)		2.5 (2)	2 (1.75)*	2 (2)*	4 (1)
Median time from symptom onset to EBP (months)		3.25 (11)	3 (3)*	9 (8)*	13 (4)

*Indicating no statistically significant difference between LP and PDA.

Of 30 eligible patients, 20 (four male and 16 female) returned the questionnaire. Mean age in this cohort was 36.4 years (± 10.6).

All 20 patients stated that their social and/or family life, sex life and work life were severely impaired by their condition. All patients reported social isolation as they were not able to attend social events anymore. All patients with partners reported that their intimate relations had suffered as well. One woman was divorced by her partner because of cPDPH.

**Figure 2:**
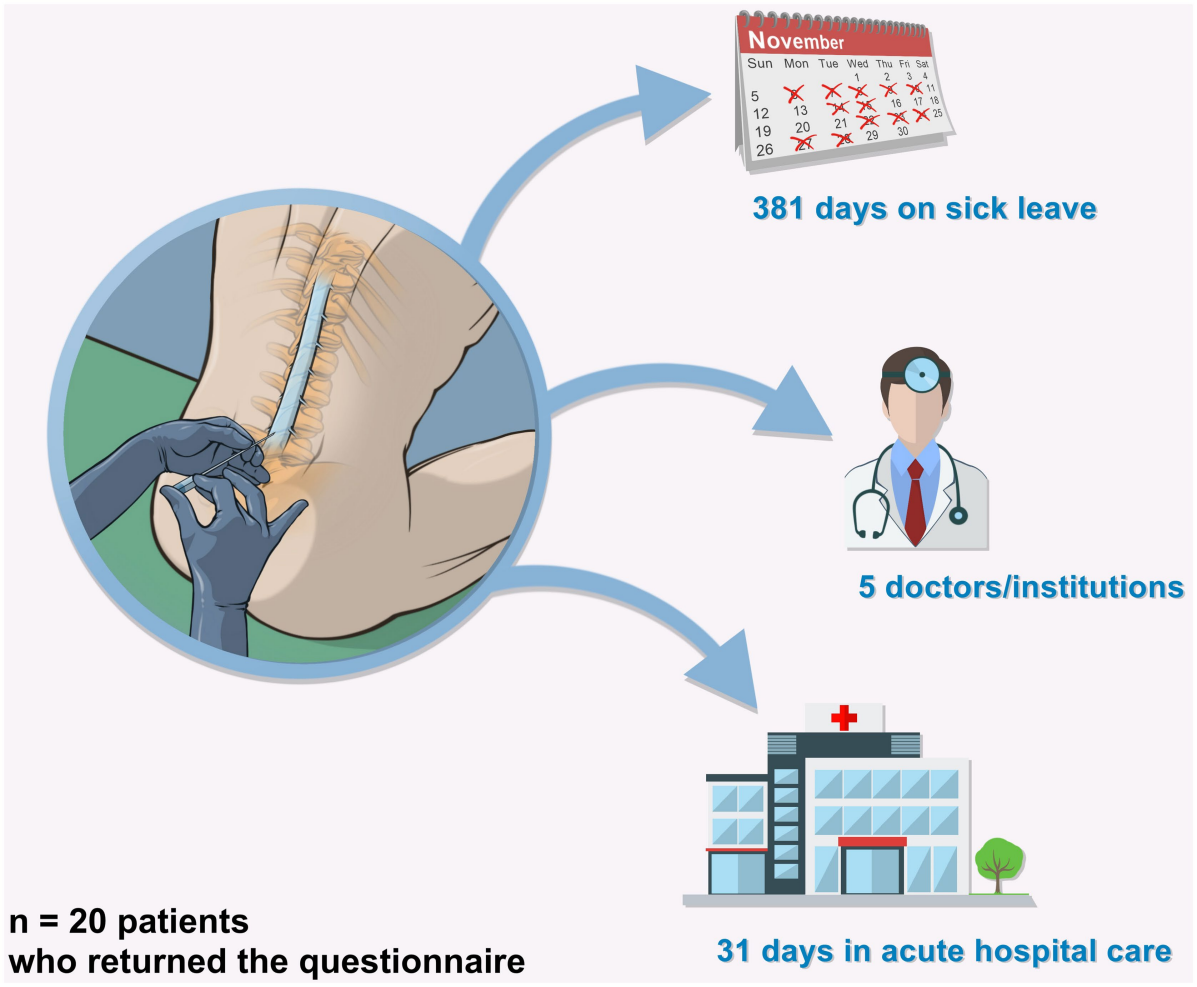
Socioeconomic impact.

In total, patients spent a median of 31 days (IQR = 20–52; min/max = 10/150) in acute hospital care and consulted a median of 5 doctors/institutions (IQR = 3.25–9.75; min/max = 1/26) due to cPDPH. Median time on sick leave was 381 days (IQR = 212–958; min/max = 14/4015) in the working population (Figure 2). Three patients became symptomatic while on parental leave and one patient was a student at the time of lumbar puncture. Three patients have not been able to return to work until the end of the follow-up. Two patients lost their jobs due to their physicial inabilities related to their. Post-dural puncture headaches. In addition, two partners of patients quit their jobs to care for the patient and/or the common children.

## Discussion

Our study demonstrates that patients with cPDPH do present with an immense individual burden of disease and profound socioeconomic impact. While these patients are in the prime of their life, many of them were on sick leave for over a year or even lost their jobs. The economic impact of a year on sick leave—in maximum 4,015 days corresponding to 11 years in one patient—is tremendous for society and for patients as they cannot provide for themselves and their families anymore. Even life-threatening complication like subdural hematomas may occur. Four out of 30 patients were diagnosed with SDH on imaging; one of them had to undergo trepenation. While a reliable analysis is not possible due to this limited patient number, one could argue that symptoms of SDH can mimic symptoms of cPDPH. Due to the retrospective character of the study and the referal bias, the current high number of patients with cPDPH in our series must be regarded with caution. Sound epidemiological data are not available.

Following a standard diagnostic procedure or a routine intervention, these patients are highly impacted by the symptoms of cPDPH and cannot continue to live a normal life. All patients clearly stated that their social life was severely impaired by cPDPH. Even intimate relations with the partner suffer which can lead to divorce as reported by one patient. Social isolation because of the inability to leave the house or even the supine position reportedly intensifies the psychological burden on these patients. At some point this may lead to comorbidities such as anxiety, depression or sleep disorders which then blend with the orthostatic symptoms.

LP, spinal anesthesia, and therapeutic interventions are routine procedures performed by different specialists. Post-dural puncture headaches are often considered a minor complication and transient phenomenon. Headaches persisting beyond that point are often neglected. However, our results demonstrate that patients suffer from prolonged headaches with major consequences. Patients are often left alone with this complication and seek help with various doctors or institutions – often on their own financial expanses – as there are no defined responsibilities. In accordance to our results, other authors have reported prolonged headaches as a complication of lumbar puncture (5, 7, 8). The probability of prolonged headache does not seem to be related to the type of procedure (9) and can affect any patient. In females, however, one should recognize PDA as a cause of cPDPH (8). Our data show that cPDPH in patients with a history of PDA does not differ from patients with LP. Finding the right diagnosis in PDA on the other hand requires a thorough workup (10). Others propose that the treatment strategy can be staggered from conservative to a more invasive approach. Conservative treatment may include bed rest, fluid therapy and painkillers followed by an epidural blood patch (3, 11) or explorative surgery. Data show that patients with post-dural puncture headache profit from early intervention by EBP (12).

While the literature agrees that PDPH can become chronic there is no widely acknowledged consensus as to when the headache may be considered chronic (13). To define chronic post-dural puncture headache some authors like us propose a duration of more than the predefined 14 days (13) while others demand one month of headaches (14). The lack of a distinct definition of cPDPH as well as the lack of an established management strategy highlights the underrated status of this condition. Due to the frequency of lumbar punctures in medical practice and the severe impact on patients, clinicians should be aware of cPDPH.

Our study is limited by its retrospective and monocentric nature as well as small patient number.

## Conclusion

Chronic post-dural puncture headache (cPDPH) puts an immense burden of disease on affected individuals, families, and the health care system. Our results imply that cPDPH needs to be considered and should be prospectively studied.

## Data Availability

The raw data supporting the conclusions of this article will be made available by the authors upon request.

## References

[1] Weir EC. The sharp end of the dural puncture. BMJ. (2000) 320:127. doi: 10.1136/bmj.320.7227.127, PMID: 10625287 PMC1117382

[2] Gobel H. (2021). ICHD-3. The international classification of headache disorders. Available at: https://ichd-3.org/ (Accessed September 24, 2021).

[3] Bezov D Ashina S Lipton R. Post-Dural puncture headache: part II – prevention, management, and prognosis. Headache J Head Face Pain. (2010) 50:1482–98. doi: 10.1111/j.1526-4610.2010.01758.x, PMID: 20807248

[4] Lavi R Yernitzky D Rowe JM Weissman A Segal D Avivi I. Standard vs atraumatic Whitacre needle for diagnostic lumbar puncture: a randomized trial. Neurology. (2006) 67:1492–4. doi: 10.1212/01.wnl.0000240054.40274.8a, PMID: 17060584

[5] Vandam LD Dripps RD. Long-term follow-up of patients who received 10,098 spinal anesthetics; syndrome of decreased intracranial pressure (headache and ocular and auditory difficulties). JAMA J Am Med Assoc. (1956) 161:586–91. doi: 10.1001/jama.1956.02970070018005, PMID: 13318967

[6] Schievink WI Maya MM Moser FG. Digital subtraction myelography in the investigation of post–dural puncture headache in 27 patients: technical note. J Neurosurg Spine. (2017) 26:760–4. doi: 10.3171/2016.11.SPINE16968, PMID: 28362213

[7] MacArthur C Lewis M Knox EG. Accidental dural puncture in obstetric patients and long term symptoms. BMJ. (1993) 306:883–5. doi: 10.1136/bmj.306.6882.883, PMID: 8490410 PMC1677341

[8] Ranganathan P Golfeiz C Phelps AL Singh S Shnol H Paul N. Chronic headache and backache are long-term sequelae of unintentional dural puncture in the obstetric population. J Clin Anesth. (2015) 27:201–6. doi: 10.1016/j.jclinane.2014.07.008, PMID: 25483233

[9] Nath S Koziarz A Badhiwala JH Alhazzani W Jaeschke R Sharma S. Atraumatic versus conventional lumbar puncture needles: a systematic review and meta-analysis. Lancet. (2018) 391:1197–204. doi: 10.1016/S0140-6736(17)32451-0 29223694

[10] van Zyl DT Klar DG. An update on effective Management of the Postdural Puncture Headache. World Federation of Societies of Anesthesiologists. (2021).

[11] Arnold MJ. Postdural puncture headache: guidelines from a multisociety. Int Working Group Am Fam Physician. (2024) 110:97–8. PMID: 39028797

[12] Kooten F Oedit R Bakker SLM Dippel DWJ. Epidural blood patch in post dural puncture headache: a randomised, observer-blind, controlled clinical trial. J Neurol Neurosurg Psychiatry. (2008) 79:553–8. doi: 10.1136/jnnp.2007.122879, PMID: 17635971

[13] Uppal V Russell R Sondekoppam R Ansari J Baber Z Chen Y. Consensus practice guidelines on postdural puncture headache from a multisociety, international working group: a summary report. JAMA Netw Open. (2023) 6:e2325387. doi: 10.1001/jamanetworkopen.2023.25387, PMID: 37581893

[14] Zhang Q Pang SY Liu CW. Chronic headaches related to post-dural puncture headaches: a scoping review. Br J Anaesth. (2022) 129:747–57. doi: 10.1016/j.bja.2022.08.004, PMID: 36085093

