# Supplementary material for: Chronic post-dural puncture headache–a serious and underrated complication following lumbar puncture: a cohort study
Source: Front Neurol. 2024 Nov 29;15:1493303. doi: 10.3389/fneur.2024.1493303 (PMC11638535; doi:10.3389/fneur.2024.1493303)
Supplement: Supplementary file 1 [file Data_Sheet_1.pdf]

Dear Patients,

You are suffering from persistent orthostatic headaches following a lumbar puncture or epidural anesthesia – a condition we refer to as "chronic post-dural puncture headache." Due to these headaches, you have been, or are currently, receiving treatment at the University Hospital Freiburg.
Despite the significant impact of this condition on both private life and health, it is often underrecognized. Therefore, we are striving to raise awareness of this condition among our medical colleagues and society as a whole. Scientific research is essential to this effort.
As part of our current work, we aim to describe the health and socioeconomic status of patients to illustrate the serious consequences of this condition.
We kindly ask you to take 10 minutes to answer the questions listed below as honestly as possible, reflecting your current state. Please complete the questionnaire in Word format, save it as a PDF, and send it back to luisa.mona.kraus@uniklinik-freiburg.de.
By participating, you are helping us immensely, and thus other affected individuals as well!

Thank you for your participation, and best regards,

Dr. Luisa Kraus
Physician
Department of Neurosurgery
University Hospital Freiburg

For any questions: luisa.mona.kraus@uniklinik-freiburg.de

Your name: Today's date:

<u>**General Questions**</u>

Date of puncture:

Reason for puncture:

Onset of symptoms:

Description of symptoms:

Total number of days in hospital care (including stay at the University Hospital Freiburg):

Total number of days unable to work due to your symptoms:

Total number of different doctors and institutions consulted due to your symptoms:

Did you incur additional financial expenses that were not covered by your health insurance?

☐ No

☐ Yes, they were:

I have received

epidural blood patches

surgeries

To date, all treatments have led to:

☐ major improvement

☐ some improvement

☐ no improvement in my symptoms.

Has your condition impacted your social life? If yes, please elaborate.

Specific HIT-6 Questionnaire

These questions are designed to help you describe and communicate how you CURRENTLY feel and what you are CURRENTLY unable to do due to headaches. Please check the answer that best applies to you for each question:

When you have headaches, how often are they severe?

☐ never ☐ rarely ☐ sometimes ☐ very often ☐ always

How often do headaches affect your daily life (e.g., household tasks, work, school, or social activities)?

☐ never ☐ rarely ☐ sometimes ☐ very often ☐ always

When you have headaches, how often do you wish you could lie down?

☐ never ☐ rarely ☐ sometimes ☐ very often ☐ always

In the past 4 weeks, how often did headaches make you feel too tired to work or perform daily activities?

☐ never ☐ rarely ☐ sometimes ☐ very often ☐ always

In the past 4 weeks, how often have you felt irritated or fed up due to headaches?

☐ never ☐ rarely ☐ sometimes ☐ very often ☐ always

In the past 4 weeks, how often did headaches limit your ability to concentrate on work or daily activities?

☐ never ☐ rarely ☐ sometimes ☐ very often ☐ always
